# Vascular aging in long-term survivors of testicular cancer more than 20 years after treatment with cisplatin-based chemotherapy

**DOI:** 10.1038/s41416-020-01049-3

**Published:** 2020-09-14

**Authors:** Johannes Stelwagen, Sjoukje Lubberts, Lars C. Steggink, Gerrie Steursma, Lara M. Kruyt, Jan Willem Donkerbroek, Arie M. van Roon, Anne I. van Gessel, Saskia C. van de Zande, Coby Meijer, Christine H. Gräfin zu Eulenburg, Sjoukje F. Oosting, Janine Nuver, Annemiek M. E. Walenkamp, Igle Jan de Jong, Joop D. Lefrandt, Jourik A. Gietema

**Affiliations:** 1 Department of Medical Oncology, University Medical Centre Groningen and University of Groningen, Groningen, The Netherlands; 2 Department of Internal Medicine, division of Vascular Medicine, University Medical Centre Groningen and University of Groningen, Groningen, The Netherlands; 3 Department of Epidemiology, University Medical Centre Groningen and University of Groningen, Groningen, The Netherlands; 4 Department of Urology, University Medical Centre Groningen and University of Groningen, Groningen, The Netherlands

**Keywords:** Testicular cancer, Outcomes research

## Abstract

**Background:**

Late effects of cisplatin-based chemotherapy in testicular cancer survivors (TCS) include cardiovascular morbidity, but little data is available beyond 20 years. The objective was to assess vascular damage in very long-term TCS.

**Methods:**

TCS (treated with chemotherapy or orchiectomy only) and age-matched healthy controls were invited. Study assessment included vascular stiffness with ultrasound measurement of carotid-femoral pulse wave velocity (cf-PWV).

**Results:**

We included 127 TCS consisting of a chemotherapy group (70 patients) and an orchiectomy group (57 patients) along with 70 controls. Median follow-up was 28 years (range: 20–42). The cf-PWV (m/s) was higher in TCS than in controls (geometrical mean 8.05 (SD 1.23) vs. 7.60 (SD 1.21), *p* = 0.04). The cf-PWV was higher in the chemotherapy group than in the orchiectomy group (geometrical mean 8.39 (SD 1.22) vs. 7.61 (SD 1.21), *p* < 0.01). In the chemotherapy group cf-PWV increased more rapidly as a function of age compared to controls (regression coefficient *b* 7.59 × 10^−3^ vs. 4.04 × 10^−3^; *p* = 0.03).

**Conclusion:**

Very long-term TCS treated with cisplatin-based chemotherapy show increased vascular damage compatible with “accelerated vascular aging” and continue to be at risk for cardiovascular morbidity, thus supporting the need for intensive cardiovascular risk management.

**Clinical trial registration:**

The clinical trial registration number is NCT02572934.

## Background

Although it accounts for only 1% of all cancers in men, testicular cancer (TC) is the most common solid malignancy affecting males between the ages of 15 and 35 years.^1^ Depending on disease stage, TC is treated by either orchiectomy alone or by orchiectomy followed by radiotherapy (RT) or platinum-based chemotherapy (CT). Since the introduction of cisplatin in the late 1970s,^2^ TC survival has increased, with 10-year survival rates reaching 90-95%.^3,4^ However, successful treatment is often accompanied by adverse late treatment effects, resulting in increased morbidity from second cancers,^5^ cardiovascular disease (CVD),^6–10^ nephrotoxicity,^11,12^ pulmonary toxicity^13^ and Raynaud’s phenomenon.^14,15^ Many of these late treatment effects of CT may be different manifestations of the same underlying vascular damage, possibly induced directly by cisplatin-based CT or indirectly by increased CVD risk factors after CT.^16,17^ This increased morbidity coincides with increased long-term mortality: patients treated with CT for TC had a 1.6-fold (95% CI = 1.0–2.5) higher risk of dying from CVD compared to the general population 10 years after treatment.^18^ An alarming clinical feature is the relatively young age at which TC survivors develop CVD,^8,10^ which led to the hypothesis that TC patients treated with CT show a phenotype resembling accelerated aging.^9,19,20^ However, the mechanisms underlying this cardiovascular morbidity have not been extensively studied.^21^ In addition, most data on the incidence of late effects have been collected through questionnaires in large epidemiological studies in TC survivors up to 15 years post-treatment. Consequently, little information is available on the health status of TC patients surviving more than 20 years. We, therefore, assessed the presence of vascular damage in this group of very long-term TC survivors in comparison to age-matched controls. Our primary aim was to quantify long-term cardiovascular morbidity and gain more insight into factors related to the development of these late treatment effects. This insight is relevant to cancer survivorship care and could guide future interventional study protocols to reduce cardiovascular morbidity in long-term testicular cancer survivors.

## Methods

### Patients

**Fig. 1:**
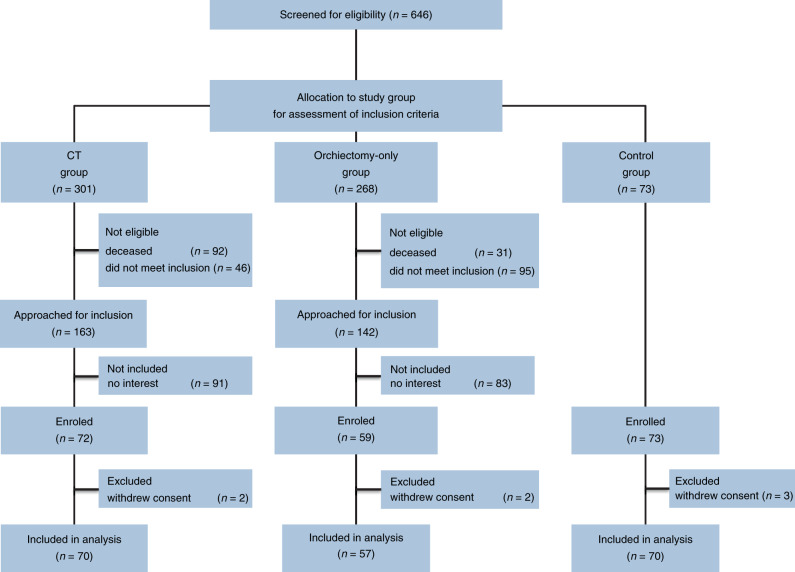
Consort diagram. The institutional database of testicular cancer patients was used to identify testicular cancer survivors (TCS) treated with chemotherapy (CT). Patients were randomly selected and screened for inclusion. Eligible CT patients were approached if they were still alive and met the inclusion criteria: <40 years of age at diagnosis, <70 years of age at inclusion in the current study, treatment for TC was ≥20 years ago and patients were treated with CT for either good or intermediate prognosis according to the International Germ Cell Consensus Classification (IGCCCG). This led to screening 301 CT patients with 138 not eligible. 91 of the eligible patients decided not to participate in the study. Next, the institutional database of testicular cancer patients was used to identify TCS treated with orchiectomy only. Patients were age-matched to the CT group and approached for inclusion if they were alive and met the inclusion criteria: <40 years of age at diagnosis, <70 years of age at inclusion in the current study, treatment for TC was ≥20 years ago and patients were not treated with CT for any indication. This led to screening 268 orchiectomy-only patients with 126 not eligible and 83 patients who decided not to participate in the study. Furthermore, 70 age-matched healthy controls were included.

In this analysis we report on two groups of TC survivors—those treated with orchiectomy only and those treated with orchiectomy followed by CT—in comparison with age-matched controls (Fig. 1). For the first group, we randomly selected 70 TC survivors from the institutional database who had previously been treated with both surgery and CT at the University Medical Centre Groningen (UMCG). For the second group, 57 age-matched patients who had been treated with orchiectomy only were included. Inclusion criteria for both groups were the following: <40 years of age at diagnosis, <70 years of age at inclusion in the current study, and treatment for TC was ≥20 years ago. For the CT group, patients were treated with CT for either good or intermediate prognosis according to the International Germ Cell Consensus Classification (IGCCCG). For both groups, patients receiving RT or CT for any other indication were excluded. The age-matched male controls were recruited through advertisement using flyers distributed in the campus area of the UMCG, especially in the non-patient areas and in neighbouring supermarkets.

### Assessments

Anthropometrics (weight, height, waist and hip circumference) and standardised blood pressure were measured. Fasting blood samples were used to establish presence of hypogonadism (defined as serum testosterone <10.9 nmol/L or treatment with testosterone supplementation), lipid profile (total cholesterol, triglycerides, high-density lipoprotein (HDL) and low-density lipoprotein (LDL)), glucose and HbA1C levels and creatinine levels. Presence of metabolic syndrome was assessed according to the National Cholesterol Education Programme (NCEP) Adult Treatment Plan (ATP) III criteria.^22^

Vascular function and structure measurements were performed by two specialised technicians at the vascular laboratory—the reproducibility was previously tested at our laboratory, showing an intraclass correlation coefficient (ICC) of 0.91 (95% CI 0.83–0.96) between the two vascular technicians.^23^ Carotid-femoral pulse wave velocity (cf-PWV)—chosen for its validated, and added value in CVD risk assessment compared to conventional risk factors alone—was calculated by simultaneously measuring arm blood pressure (SphygmoCor). Measurements were performed in duplicate and single sided for each patient (a description in more detail is provided in Supplementary Methods). Only measurements with a variance of <10% were considered reliable. All measurements were performed according to the quality guidelines of the SphygmoCor. Presence of Raynaud’s phenomenon, as a potential sign of small vessel disease, was evaluated with standardised digital cooling tests (fingertip photoelectric plethysmography (PPG), cooling from room temperature to 6 °C). Symptoms of Raynaud’s phenomenon were scored with the Scale for Chemotherapy-Induced Neurotoxicity (SCIN).^24^

Fasting blood samples were used to assess biochemical markers for vascular damage (von Willebrand factor (vWF)), coagulation markers (i.e. FVIII, fibrinogen, plasminogen activator inhibitor-1 (PAI-1) antigen and tissue plasminogen activator (tPA)) and C-reactive protein (CRP). To assess creatinine clearance and albuminuria, 24-h urine samples were used.

### Statistical analysis

cf-PWV was skewed and was log transformed to approximate a normal distribution. Differences in cf-PWV are reported as geometrical means. Multivariate regression analysis was performed on the logarithmic transformed cf-PWV according to a stepwise backwards method. Corresponding regression coefficients and p-values are reported. Plotting cf-PWV as a function of age was done by using the previously calculated regression coefficients, transformed back to a non-logarithmic value. Regression coefficients of age and cf-PWF were compared by including the interaction term of [age*treatment group] in the multiple regression model, and corresponding P-values for the difference in slopes are reported. Additional statistical methods are described in the supplementary Patients and Methods. All *P*-values were two sided and the threshold of statistical significance was set at *p* < 0.05. The data were analysed by using SPSS 23.0 (IBM-SPSS, Chicago, IL, USA).

## Results

From August 2015, we included 70 TC survivors treated with orchiectomy and CT (CT group), 57 treated with orchiectomy only and 70 age-matched controls (Fig. 1). Patient demographic, clinical and laboratory characteristics at follow-up according to treatment group are reported in Table 1. Additional patient characteristics including details on chemotherapeutic regimens used and medical history of cardiovascular disease are shown in Table 2. There were no significant differences in age, follow-up (FU) duration, smoking behaviour, pack years, renal function, serum glucose and HbA1C between TC survivors and the controls. TC survivors were more likely to have hypertension compared to the controls (OR 2.2 [95% CI, 1.2–4.0]). The prevalence of hypertension was comparable for the CT group and the orchiectomy only group. TC survivors had higher BMI compared to the controls (median 26.5 kg/m^2^ [range 20–42] vs. 25.6 kg/m^2^ [range 20–38], *p* = 0.02). Use of lipid-lowering medication was more prevalent in TC survivors compared to controls (OR 3.0 [95% CI, 1.1–8.1], *p* = 0.03) and was more prevalent in the CT group than in the orchiectomy-only group (OR 3.2 [95% CI, 1.2–8.6], *p* = 0.02). Total levels of testosterone were lower in TC survivors compared to controls (median 13.5 [range 2.9–35.8] vs. 15.4 [range 7.2–46.0], *p* = 0.01). Hypogonadism was more prevalent in the CT group compared to the orchiectomy-only group (12% vs. 2%, *p* = 0.03).

**Table 1:** Patient demographic, clinical, and laboratory characteristics at follow-up according to study group.

	Testicular cancer survivors (TCS)							Healthy controls		
	All TCS		CT		Orchiectomy only		CT vs. Orchiectomy only	Controls		TCS vs. Controls
Characteristic	*n* = 127		*n* = 70		*n* = 57			*n* = 70		
	No.	%	No.	%	No.	%	*P*-value	No.	%	*P*-value
Age, years										
At diagnosis										
Median	27		28		27		0.93	–		NA
Range	17–46		17–46		17–45			–		
At study visit										
Median	57		56		57		0.95	57		0.73
Range	40–70		41–70		40–70			39–70		
FU duration, years										
Median	28		27		28		0.74	–		NA
Range	20–42		20–40		20–42			–		
Clinical stage^†^										
I	57	45	–	–	57	100	<0.01	–		NA
II	41	32	41	59	–	–		–		
III	7	6	7	10	–	–		–		
IV	22	17	22	31	–	–		–		
Smoking behaviour										
Never smoked	56	44	29	41	27	47	0.37	37	53	0.45
Former smoker	51	40	32	46	19	33		26	37	
Current smoker	20	16	9	13	11	19		7	10	
Pack years^‡^										
Median	12		11		16		0.48	13		0.71
Range	1–48		1–48		1–44			0–42		
GFR, ml/min^§^										
Mean	129		125		133		0.15	135		0.17
SD	32		35		26			31		
Blood pressure, mmHg										
Systolic										
Mean	133		132		133		0.75	133		0.81
SD	14		13		14			16		
Diastolic										
Mean	87		89		87		0.38	87		0.79
SD	9		9		10			11		
Hypertension^¶^	88	69	51	73	37	65	0.22	34	52	0.01
BMI, kg/m²										
Median	26.5		25.9		26.9		0.05	25.6		0.02
Range	21–42		21–36		21–42			20–38		
Hip circumference (cm)										
median	102		99		106		<0.001	99		<0.001
range	90–124		90–124		95–119			85–119		
Waist circumference (cm)										
Median	98		98		98		0.53	95		0.004
range	77–144		80–126		77–144			76–133		
Obesity										
25-30	54	43	27	39	27	47	0.32	21	30	0.68
>30	16	13	7	10	9	14		7	10	
Total cholesterol										
Mean	5.4		5.5		5.3		0.23	5.5		0.47
SD	1.0		0.9		1.2			1.0		
Lipid-lowering medication	25	20	19	27	6	11	0.02	5	7	0.03
Serum glucose, mmol/L										
Median	5.8		5.8		5.8		0.21	5.8		0.40
Range	4.7–15.1		4.7–9.3		5.0–13.8					
HbA1c, %										
Median	5.5		5.5		5.5		0.42	5.4		0.17
Range	4.5–11.3		4.5–11.3		4.6–7.4			4.8–8.0		
Known Diabetes Mellitus	3	2	2	3	1	2	0.68	1	3	0.64
Metabolic syndrome^#^	33	26	16	23	17	30	0.40	11	16	0.15
Total testosterone, nmol/L										
Median	13.5		12.2		14.2		0.28	15.4		0.01
Range	2.9–35.8		2.9–26.5		5.6–35.8			7.2–46.0		
Serum LH, U/L										
Median	8.0		10.5		7.3		<0.01	4.8		<0.01
Range	2.9–47.0		2.0–46.6		2.9–47.0			1.3–12.4		
Testosterone therapy^††^	9	7	8	12	1	2	0.03	–	–	0.03
Hypogonadism	21	17	14	20	7	12	0.23	2	3	<0.01

*TCS* testicular cancer survivors, *CT* chemotherapy group, *FU* follow-up, *GFR* glomerular filtration rate, *SD* standard deviation, *BMI* body mass index, *HbA1c* glycated haemoglobin, *LH* luteinising hormone.

^†^According to Royal Marsden classification.

^‡^Including only smokers.

^§^Measured through two 24-h urine samples.

^¶^Hypertension defined as systolic >140 mmHg and/or diastolic >90 mmHg or use of antihypertensive medication.

^#^Definition according to NCEP ATP III criteria.

^††^Using testosterone for previously diagnosed hypogonadism.

^‡‡^Hypogonadism defined as either using testosterone therapy for previously diagnosed hypogonadism or serum testosterone <8 nmol/L.

**Table 2:** Supplementary patient characteristics including chemotherapy regimens and medical history of cardiovascular disease.

Treatment	No. of patients	%	Total no. of cycles	
Chemotherapy regimens				
BEP	19	28	4†	
EP	14	20	4	
BEP + EP^‡^	9	13	4	
PVB	12	17	4	
BEP/PVB^§^	9	6	4	
BEP + VIP	4	6	6	
VIP	3	4	4	
Cardiovascular events (in CT)			Year of diagnosis	Regimens used
AMI	3(3)	2	2006, 2014, 2017	EP (2x), BEP
PE	3(2)	2	1993, 1998, 2012	BEP, BEP/VIP
PAD	–	–		
CVA	3(2)	2	1994, 2004 (2x)	PVP (2x)
DVT	5(3)	4	1995 (2x), 2006, 2015 (2x)	EP, BEP, PVB

*BEP* bleomycin, etoposide, cisplatin, *EP* etoposide, cisplatin, *PVB* cisplatin, vinblastin, bleomycin, *Vbl* vinblastin, *VIP* etoposide, ifosfamide, cisplatin, *CT* chemotherapy group, *AMI* acute myocardial infarction, *PE* pulmonary embolism, *PAD* peripheral arterial disease, *CVA* cerebrovascular accident, *DVT* deep venous thrombosis.

^†^One patient received three cycles of BEP chemotherapy.

^‡^Patients received three cycles of BEP chemotherapy followed by 1 cycle of EP chemotherapy.

^§^Patients received alternating BEP and PVB chemotherapy regimens.

### Arterial stiffness

**Fig. 2:**
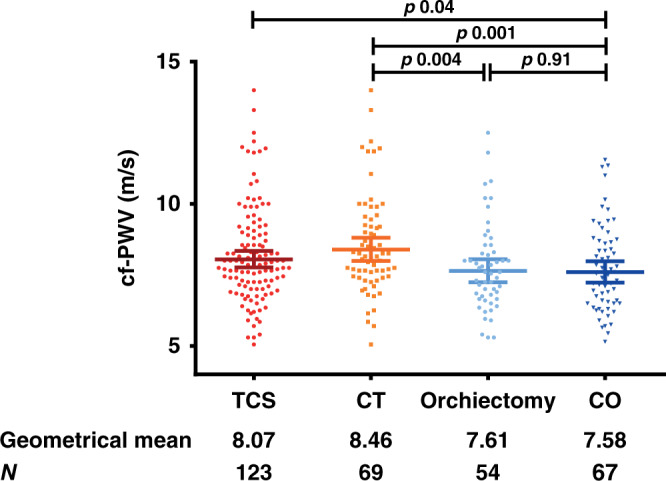
Pulse wave velocity (cf-PWV) for all TC survivors (TCS), controls (CO) and for separate study groups. Pulse wave velocity (cf-PWV) as biomarker for vascular stiffness for all TC survivors (TCS), controls (CO) and for separate study groups. Bars represent median values and interquartile range. Corresponding geometrical means are reported. P values were obtained by students T-test of the logarithmic transformation.

**Fig. 3:**
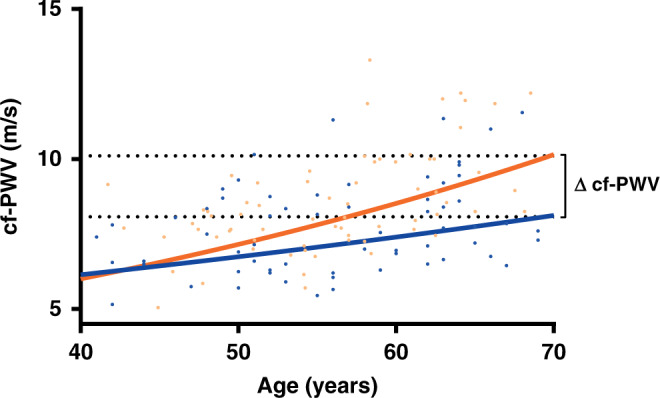
Pulse wave velocity (cf-PWV) as biomarker for vascular stiffness as a function of age for the chemotherapy (CT) group and the control group. Orange line represents the CT group and blue line the control group. Corresponding linear regression lines were based on regression coefficients for age calculated in the multivariate model correcting for other predictive variables. ∆cf-PWV at age 70 amounts to 2.03 m/s. For CT: 10^(0.475 + (7.590 × 10–3 × Age))^. For CO: 10^(0.627 + (4.035 ×10–3 × Age))^. Slopes differ significantly (*p* = 0.03).

cf-PWV measurements were available for 190 of 197 participants—seven measurements were excluded due to variance above 10%—and are depicted in Fig. 2. TC survivors had higher cf-PWV compared to controls (geometrical mean 8.07 m/s [95% CI: 7.82–8.37] vs. 7.58 m/s [95% CI: 7.24–7.94] *p* = 0.04). The CT group had higher cf-PWV compared to the orchiectomy-only group (geometrical mean 8.46 m/s [95% CI: 8.06–8.88] vs. 7.61 m/s [95% CI: 7.22–8.02], *p* = 0.004). There was no difference in cf-PWV between the orchiectomy-only group and controls (geometrical mean 7.61 m/s [95% CI; 7.22–8.02] vs. 7.58 m/s [95% CI; 7.24–7.94], *p* = 0.91). The cf-PWV was comparable for different chemotherapy regimens used in the CT group. In a multiple regression analysis on cf-PWV including all participants, age, presence of metabolic syndrome, having received CT and the amount of smoking pack years were independent factors (model R 0.67, *p* < 0.01, Table 3). Other factors included in the model, such as BMI and testosterone levels, did not significantly contribute to the model. cf-PWV as a function of age using the regression coefficients calculated in the multivariate model is depicted in Fig. 3 for participants in the CT group and control group. The linear regression line of cf-PWV and age had a steeper slope in the CT group compared to the control group (regression coefficient *b* 7.59 × 10^−3^; standard error (SE) 1.22 × 10^−3^ vs. 4.04 × 10^−3^; SE 1.23 × 10^−3^, *p* = 0.03). Regression formulas and comparison of regression coefficients of the groups are shown in Supplementary Table 1.

**Table 3:** Explaining variance of cf-PWV: multiple regression models for ^10^log [cf-PWV] per study groups.

Study groups	Standardised coefficients		*P*-value	Model R
	*β*	SE		
All				0.67
Constant	0.541	0.036	<0.01	
Age	5.638 × 10^−3^	0.951 × 10^−3^	<0.01	
Metabolic syndrome (Y/N)	6.780 × 10^−2^	1.909 × 10^−2^	<0.01	
Smoking (pack years)	1.077 × 10^−3^	0.752 × 10^−3^	0.015	
Chemo (Y/N)	4.402 × 10^−2^	1.019 × 10^−2^	<0.01	
TCS				0.72
Constant	0.492	0.043	<0.01	
Age	6.508 × 10^−3^	0.761 × 10^−3^	<0.01	
Metabolic syndrome (Y/N)	5.999 × 10^−2^	1.361 × 10^−2^	<0.01	
Smoking (pack years)	0.973 × 10^−3^	0.502 × 10^−3^	0.055	
Chemo (Y/N)	4.705 × 10^−2^	1.178 × 10^−2^	<0.01	
CT				0.66
Constant	0.475	0.070	<0.01	
Age	7.590 × 10^−3^	1.220 × 10^−3^	<0.01	
Metabolic syndrome (Y/N)	6.079 × 10^−2^	2.006 × 10^−2^	<0.01	
Smoking (pack years)	1.272 × 10^−3^	0.684 × 10^−3^	0.07	
Orchiectomy only				0.78
Constant	0.559	0.053	<0.01	
Age	5.353 × 10^−3^	0.951 × 10^−3^	<0.01	
Metabolic syndrome (Y/N)	7.386 × 10^−2^	1.909 × 10^−2^	<0.01	
Smoking (pack years)	0.233 × 10^−3^	0.752 × 10^−3^	0.76	
Controls				0.55
Constant	0.627	0.069	<0.01	
Age	4.035 × 10^−3^	1.230 × 10^−3^	<0.01	
Metabolic syndrome (Y/N)	8.319 × 10^−2^	2.520 × 10^−2^	<0.01	
Smoking (pack years)	1.657 × 10^−3^	0.875 × 10^−3^	0.06	

Multiple regression models per study group with standardised coefficients (β) and standard errors (SE). Positive β corresponds to a positive correlation. Proportion of variation in cf-PWV explained by each model is shown as R (0.55–0.78). Models based on the logarithmic transformed cf-PWV. Note that receiving chemo is an independent predictor when the total study population was modelled as well as when only TCS were modelled. Furthermore, note the difference in coefficients β for age in the CT group and control group.

### Raynaud’s phenomenon

**Fig. 4:**
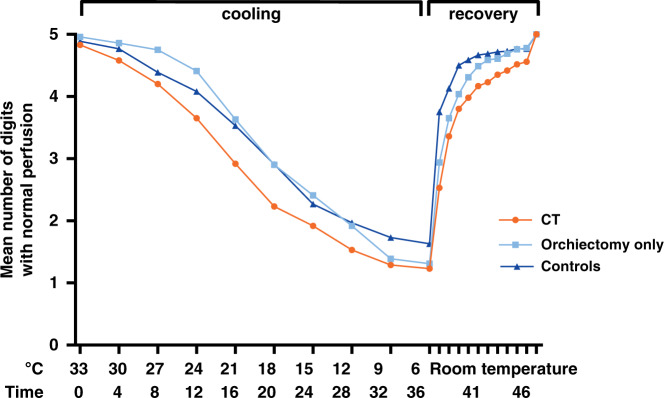
Raynaud Phenomenon tested by cooling digits. Mean number of digits with perfusion at each time point during PPG-test. The first 36 min represent the cooling period. Next, 10 min of recovery time at room temperature is shown. Mean ischaemic and recovery times for each study group are reported in Supplementary Table 3.

Compared to 11% in the control group, 29% of TC survivors reported clinically significant symptoms of Raynaud’s phenomenon (*p* = 0.004). Patients in the CT group reported symptoms more frequently than those in the orchiectomy-only group (41% vs. 16%, *p* = 0.002). In the CT group, 22% had a high cumulative symptom score compared to 4% in the surgery group (*p* = 0.003) and 6% in the control group (*p* = 0.006) (Supplementary Fig. 1). The mean number of digits with normal perfusion at any point in time during PPG is depicted in Fig. 4. Ischaemic time was similar in TC survivors (median 15.0 min. [range 0.0–37.6]) and in controls (median 12.6 min., range 0.0–43.4, *p* = 0.09). Patients in the CT group had a longer ischaemic time (median 18.3 min. [range 0.0–37.6]) compared to controls (median 12.6 min. [range 0.0–43.4], *p* = 0.01) and to the orchiectomy-only group (12.4 min. [range 0.0–31.8], *p* = 0.02). Recovery time was longer in TC survivors (median 1.0 min. [range 0.0–10.0]) compared to controls (median 0.0 min. [range 0.0–9.6], *p* < 0.001). Patients in the CT group had a similar recovery time (median 1.4 min. [range 0.0–10.0] vs. 0.6 min. [range 0.0–7.2], *p* = 0.15) to those in the orchiectomy-only group (Supplementary Table 2). Symptoms correlated with recovery time on PPG (β 0.32, *p* < 0.001). We found no significant relationship between Raynaud’s phenomenon and cf-PWV in the CT group.

### Vascular biomarkers

Median values of biochemical markers for vascular damage, including vWF, presence of albuminuria, coagulation markers (fibrinogen, PAI-1, tPA and F-VIII)) and CRP are listed in Table 4. Albuminuria occurred more often in TC survivors than in the control group (OR 8.9; 95% CI, 2.0–38.6; *p* < 0.01). cf-PWV was higher in participants with albuminuria compared to those without (geometrical mean 8.90 m/s [95% CI, 8.27–9.59] vs. 7.74 m/s [95% CI, 7.50–7.99], *p* = 0.001). Levels of fibrinogen, PAI-1, tPA-antigen and CRP were higher in TC survivors than in the control group, but vWF and F-VIII levels did not differ (Table 3). No differences were found between the CT group and orchiectomy-only group. Fibrinogen, PAI-1 and tPA-antigen levels moderately correlated with cf-PWV in univariate regression (Spearman’s rho 0.21 [*p* = 0.005], 0.37 [*p* < 0.001] and 0.34 [*p* = 0.001], respectively).

**Table 4:** Biochemical markers for vascular damage per treatment group.

	Testicular cancer survivors (TCS)							Healthy controls		
	All TCS		CT		Orchiectomy only		CT vs. Orchiectomy only	Controls		TCS vs. controls
Characteristic	*n* = 126		*n* = 69		*n* = 57			*n* = 65		
	No.	%	No.	%	No.	%	*P*-value^†^	No.	%	*P*-value^†^
vWF										
median	107		110		106		0.78	96		0.16
range	35–256		59–250		35–256			32–208		
No Albuminuria	96	78	50	72	46	85	0.09	63	97	<0.01
Albuminuria^‡^	27	22	19	28	8	15		2	3	
Fibrinogen										
median	3.0		3.0		3.1		0.70	2.8		<0.01
range	1.8–6.1		1.8–5.3		2.0–6.1			1.9–4.0		
PAI-1										
median	31		35		26		0.59	23		<0.01
range	8–151		12–149		8–151			7–87		
tPA-antigen										
median	14		15		13		0.90	12		<0.01
range	4–75		4–26		5–75			4–24		
F-VIII										
median	163		160		167		0.40	157		0.81
range	11–536		81–316		11–536			75–281		
CRP										
median	1.4		1.3		1.6		0.26	1.0		0.047
range	0–45		0–29		0–45			0–13		

*vWF* Von Willebrand Factor, *PAI-1* plasminogen activator inhibitor-1, *tPA* tissue plasminogen activator, *F-VIII* factor VIII, *CRP* C-reactive protein.

^†^Mann–Whitney U test for continuous variables, χ2 for dichotomous variable.

^‡^Defined as either micro-albuminuria or macro-albuminuria. Micro-albuminuria defined as 20–200 mg/L urine and macro-albuminuria as >200 mg/L. Measured by 24-h urine samples.

## Discussion

In this uniquely long-term follow-up study focussing on vascular aging in TC survivors, median follow-up duration was 28 years. Signs of vascular damage were present in several compartments, illustrated by increased vascular stiffness measured by cf-PWV, increased ischaemic- and recovery time during digital cooling tests and the presence of albuminuria. The CT group had increased cf-PWV compared to the orchiectomy-only group and healthy controls. In a multivariate regression model including classical vascular risk factors, such as age, lipid levels, presence of hypertension and smoking habits, CT was a significant independent predictor for increased cf-PWV. The slope of the curve for cf-PWV as a function of age was significantly steeper in the CT group than in the control group, also when other known predictors of cf-PWV were taken into account such as smoking and components of metabolic syndrome.

The above findings on vascular stiffness support the notion of accelerated vascular aging after CT in TC survivors.^25^ The clinical relevance of increased arterial stiffness has become increasingly apparent by its proven added value in CVD risk assessment compared to conventional risk factors alone (e.g. age, sex, blood pressure, total cholesterol, high-density lipoprotein cholesterol, smoking status and diabetes) improving 10-year CVD risk prediction up to 13%.^26^ Consequently, assessment of arterial stiffness is currently recommended in guidelines to improve CVD risk stratification.^25^ A systematic review and meta-analysis of more than 15,000 subjects showed that an increase of cf-PWV by 1 m/s corresponded to an age-, sex- and conventional risk factor-adjusted risk increase of 14% for CVD after a mean follow-up of 7.7 years.^26,27^ As shown in Fig. 3, the assumption of accelerated vascular aging in our study led to a difference in cf-PWV between the CT group and healthy controls of 2.03 m/s at age 70. This difference would correspond with an increase in relative risk of 30.5% for cardiovascular events after a mean follow-up period of 7.7 years. This seems in some contrast with the finding of a normalised CVD mortality ratio five years after diagnosis in 15.000 TC patients registered in the Surveillance, Epidemiology, and End Results (SEER) database.^28^ However, evidently earlier after treatment and—as the authors suggest—their results might be attenuated by improved CVD management. Furthermore, they reported on cardiovascular mortality rather than on CVD prevalence. Clinical data from the Danish Testicular Cancer database, confirmed a normalised mortality ratio after one year, but found a 1.6-fold (95% CI = 1.0–2.5) higher risk of dying from CVD compared to the general population longer (10 years) after treatment.^18^ Several hypotheses on the potential mechanisms behind this increased cardiovascular morbidity with an early and a late peak in prevalence have been suggested. For one, direct vascular damage of cisplatin-based chemotherapy could increase early cardiovascular events and the vascular age at time of treatment, only to become symptomatic after years of additional aging.^17,29^ Secondly, cisplatin-based chemotherapy might cause an increased prevalence of known CVD risk factors, thus indirectly resulting in subclinical cardiovascular damage and CVD in TC survivors earlier than expected.^17^ Both these mechanisms—potentially in combination responsible—might be further worsened by long-term circulating platinum, detectable up to 20 years after administration of cisplatin-based chemotherapy.^30^ Therefore, high-risk populations such as TC survivors treated with platinum-based CT could benefit from more intensive CVD risk factor management in order to reduce long-term cardiovascular morbidity. Interventions such as exercise training have repeatedly shown efficacy in preventing and reversing age-related arterial stiffness in patients with vascular risk factors,^31–33^ and its clinical benefit in the oncological setting is still being explored (NCT01642680). Meanwhile, prospective data of studies aiming to reduce the prevalence of the metabolic syndrome in TC survivors, another important determinant of cf-PWV and CVD, are awaited. As intervention, two randomised controlled trials are currently recruiting TC patients with low serum testosterone post treatment randomising between testosterone supplementation or placebo in order to reduce the risk of metabolic syndrome (NCT02991209 and NCT03339635) which could concurrently reduce CVD risk.

In line with the increased ischaemic and recovery time, the CT group in our study reported symptoms of Raynaud’s phenomenon more often compared to the orchiectomy-only group and controls (41%). Previously reported incidences range between 20% and 40%, which is comparable to our findings.^14,15,34^ However, in our study cf-PWV was not correlated with symptoms of Raynaud’s phenomenon or with ischaemic and recovery time during PPG. Therefore, these vascular phenomena may not be the result of the same pathophysiologic mechanism. In contrast, we showed that increased prevalence of albuminuria in TC survivors correlated with cf-PWV and could, therefore, be a manifestation of a common systemic vascular pathology. This is supported by elevated vascular biomarkers that also correlated with cf-PWV. Although these vascular biomarkers were statistically not different between the CT group and the orchiectomy only group (Table 4), this can partly be explained by a limited statistical power—CT treated survivors showed a trend towards higher vascular biomarkers. On the other hand, it might be the case that orchiectomy only is capable of its own to induce endothelial activation through a change in metabolic balance.^35^ Another study showed that independent risk factors for albuminuria overlapped with those for both CVD and cf-PWV (e.g. age, obesity and components of metabolic syndrome).^36^ Therefore, interventions to address cf-PWV and risk factors for CVD may also reduce the prevalence of albuminuria in TC survivors, regardless of initial treatment modality.

Regarding possible limitations of this study: due to the single-cohort design with patients who have survived after TC treatment for a long period of time, our results could be influenced by selection bias. We lack data from deceased patients, who might have died early from CT-related causes, both during treatment and earlier during follow-up. The high proportion (27% in CT group) of patients on lipid-lowering medication could also attenuate the differences in cf-PWV reported. Consequently, our results potentially underestimate the burden of vascular damage in TC survivors. The relatively low participation rate of 55% could also have biased our results. Also, 23% of the CT survivors were treated with the PVB regimen—some of which received maintenance cisplatin treatment—potentially partly limiting this study’s conclusion to currently treated TC patients.

In conclusion, TC survivors treated with CT show features of accelerated vascular aging as shown by increased arterial stiffness. This coincides with other vascular damage parameters such as albuminuria. cf-PWV is an important determinant in the development of CVD. We therefore advocate more intensive CVD management in TC survivors, especially when treated with platinum-based combination CT. This should start early at initiation of treatment, should continue at least one year after completion of treatment and preferably continue beyond the ten-year follow-up duration as currently recommended. Determining cf-PWV in TC survivors could aid in identifying survivors at risk for future CVD development. Prospective data from interventions, such as prevention or treatment of metabolic syndrome, shortly after treatment or during follow-up will also help develop an optimal strategy to ameliorate the accelerated aging process observed in TC survivors.

## Supplementary information

Supplementary material

## Data Availability

The datasets generated and/or analysed during the current study
