# Supplementary material for: Vascular aging in long-term survivors of testicular cancer more than 20 years after treatment with cisplatin-based chemotherapy
Source: Br J Cancer. 2020 Sep 14;123(11):1599–607. doi: 10.1038/s41416-020-01049-3 (PMC7686327; doi:10.1038/s41416-020-01049-3)
Supplement: Supplementary file 1 — Supplementary material [file 41416_2020_1049_MOESM1_ESM.docx]

**Supplementary material.**

**Supplementary methods** Detailed description of assessment regarding carotid-femoral pulse wave velocity, fingertip photoelectric plethysmographies and symptoms of Raynaud’s phenomenon. Additional statistical methods are described.

**Supplementary Figure 1** Proportion of participants reporting clinically relevant complaints of Raynauds phenomenon per study group.

**Supplementary Table 1** Regression formulas for ^10^log [cf-PWV] as a function of age.

**Supplementary Table 2** Ischemic and recovery times assessing Raynaud’s phenomenon per treatment group.

**Supplementary Patients and Methods**

Assessments

Carotid-femoral pulse wave velocity (cf-PWV) was measured using the SphygmoCor Cardiovascular Management Suite (CvMS) device [Atcor Medical, Sydney]. This device uses applanation tonometry for acquisition of the carotid and femoral blood pressure waveforms. This is done sequentially, allowing a single operator to acquire the measurements. The transit time from the foot of the R-wave of the simultaneously acquired electrocardiogram of the carotid and femoral pulse is measured. The difference between these 2 transit times is divided by distances measured from the body surface to estimate the arterial path length in order to calculate the cf-PWV. The distance on the body surface is measured through subtraction of suprasternal notch-to-carotid distance from the suprasternal notch-to femoral distance. This methodology for cf-PWV is reproducible, with a within-observer variability of 0.07 ± 1.17 m/s and a between-observer variability of 0.30 ± 1.25 m/s, and has been validated against invasive measurement of aortic PWV (Butlin & Qasem, 2016).

Fingertip photoelectric plethysmographies (PPGs) were analysed by calculating ischemic time and recovery time. Ischemic time is defined as time (minutes) between loss of digital perfusion and start of recovery period. Recovery period starts when cooling stops, either after 36 minutes or when the participant requests to stop. Recovery time was defined as time (minutes) between start of recovery period and recovered normal digital perfusion. Symptoms of Raynaud’s phenomenon were with the Scale for Chemotherapy-Induced Neurotoxicity: By convention, an item score of ≥ 2 identified a clinically significant symptom. Single items were summed to give a possible score between 0–6. The subscale scores were used to allocate TC survivors into low-score groups (sum score ≤3) and high-score groups (sum score ≥4).

Statistical analysis

Differences between continuous baseline characteristics were assessed by using unpaired students T test or Mann-Whitney U test, depending on Gaussian distribution. For categorical variable, differences were assessed using χ2 tests. For normally distributed variables, mean values and corresponding standard deviations are reported. For non-Gaussian distributed variables, median values and full range are reported. For dichotomous variables odds ratios (OR) and 95% confidence intervals (CI) are presented. Individuals who received testosterone supplementation were excluded from testosterone analysis.

**Supplementary Figure 1:** Proportion of participants reporting clinically relevant complaints of Raynauds phenomenon per study group. P-values were obtained with an χ^2^-test. Striped part of each bar represents the proportion of participants with a high cumulative symptom score. The testicular cancer survivors (TCS) consist of 126 patients. The chemotherapy group consists of 69 patients, the orchiectomy only group of 57 patients. The control group consists of 70 participants.


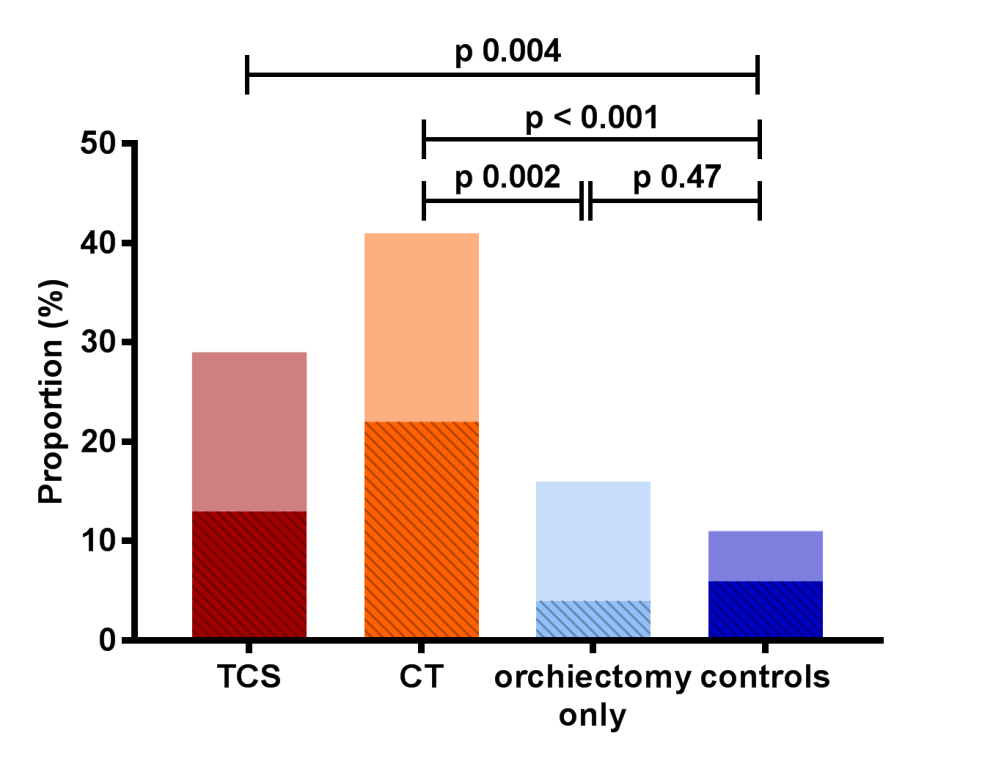


| Supplementary table 1. Regression formulas for ^10^log [cf-PWV] as a function of age | | | |
| --- | --- | --- | --- |
| **Groups** | **Regression formula** | **P-value†** | |
| **Controls** | 10^0.627 + (4.035 x 10 ̄³ x^ *^AGE^*^)^ | reference | - |
| **TCS** | 10^0.492 + (6.508 x 10 ̄³ x^ *^AGE^*^)^ | 0.08 | - |
| **CT** | 10^0.475 + (7.590 x 10 ̄³ x^ *^AGE^*^)^ | 0.03 | reference |
| **Orchiectomy only** | 10^0.559 + (5.353 x 10 ̄³ x^ *^AGE^*^)^ | 0.49 | 0.16 |
| Legend: For each study group the regression formulas with age as the predicting variable is reported. Standardised coefficients were obtained through multivariate regression models reported in Table 2. P-values obtained by adding the interaction term [study group * age] in the multivariate regression model. First column shows regression comparison of regression coefficients for age between controls and other study groups. Second column shows comparison of regression coefficient between the CT and orchiectomy only group. | | | |
|  |  |  |  |
|  |  |  |  |
|  |  |  |  |
|  |  |  |  |
|  |  |  |  |
|  |  |  |  |

| Supplementary table 2. Ischemic and recovery times assessing Raynaud’s phenomenon per treatment group | | | | | | | | | | |
| --- | --- | --- | --- | --- | --- | --- | --- | --- | --- | --- |
|  | **Testicular cancer survivors (TCS)** | | | | | |  | **Healthy controls** | |  |
|  | **All TCS** | | **CT** | | **Orchiectomy only** | | **CT vs.** | **controls** | | **TCS vs.** |
|  | **N = 126** | | **N = 69** | | **N = 57** | | **Orchiectomy** | **N = 70** | | **controls** |
| **Characteristic** | No. % | | No. % | | No. % | | **P-value**† | No. % | | **P-value**† |
| Ischemic time |  | |  | |  | |  |  | |  |
| median | 15.0 | | 18.3 | | 12.4 | | 0.02 | 12.6 | | 0.09 |
| range | 0.0 – 37.6 | | 0.0 – 37.6 | | 0.0 – 31.8 | |  | 0.0 – 43.4 | |  |
| Recovery time |  | |  | |  | |  |  | |  |
| median | 1.0 | | 1.4 | | 0.6 | | 0.15 | 0.0 | | < 0.001 |
| range | 0.0 – 10.0 | | 0.0 – 10.0 | | 0.0 – 7.2 | |  | 0.0 – 9.6 | |  |
| Symptoms |  |  |  |  |  |  |  |  |  |  |
| clinically relevant | 37 | 29 | 28 | 41 | 9 | 16 | 0.002 | 8 | 11 | 0.004 |
| high cumulative | 17 | 13 | 15 | 22 | 2 | 4 | 0.003 | 4 | 6 | 0.09 |
| † Mann-Whitney-U-test for continuous variables, χ2 for dichotomous variable. | | | | | | | | | | |
